# Multiple Factors Influencing Healthy Performance for Pre-professional and Professional Classical Violinists: An Exploratory Study Focusing on Physical Health

**DOI:** 10.3389/fpsyg.2022.791339

**Published:** 2022-05-23

**Authors:** Suze Steemers, Mario Veen, Marienke van Middelkoop, Sita M. A. Bierma-Zeinstra, Janine H. Stubbe

**Affiliations:** ^1^Codarts Rotterdam, University of the Arts, Rotterdam, Netherlands; ^2^Department of General Practice, Erasmus MC University Medical Center Rotterdam, Rotterdam, Netherlands; ^3^Performing artist and Athlete Research Lab (PEARL), Rotterdam, Netherlands; ^4^Rotterdam Arts and Sciences Lab (RASL), Rotterdam, Netherlands

**Keywords:** healthy performance, violinist, conservatoire, orchestra, health education, exploratory research

## Abstract

Musculoskeletal complaints are common in pre-professional and professional classical violinists and these complaints can affect violinists’ performance. Therefore, it is important to identify the factors that contribute to healthy performance in this population. Qualitative studies with a variety of stakeholders are able to provide insights from different perspectives into factors influencing healthy performance for the pre-professional and professional classical violinist. In the current small-scale, exploratory study, semi-structured interviews were conducted with various stakeholders; two classical violin students, one classical violin teacher, a physiotherapist, a professional classical violinist, who is also a performance coach, and a health specialist who also graduated as a professional classical violist. Thematic analysis was conducted using Atlas.ti 9. We identified six themes that were indicated as important by the participants in terms of ensuring healthy performance for the pre-professional and professional classical violinist. The themes were: (1) physical aspects (involved in playing the violin); (2) practice routine and techniques; (3) interaction between physical and mental aspects; (4) culture; (5) role of the main subject teacher; and (6) preventive measures. Furthermore, when asked specifically about the development of a physical screening tool, the participants indicated that such a tool should include multiple factors covering various regions of the body, the inclusion of a questionnaire on risk-factors, and follow-up measurements. Also, collaborations between health professionals and main subject teachers were recommended as part of the screening tool to increase commitment of participating students. The results of the current study are based on the opinions, attitudes, and ideas of a small, selected group of participants only and cannot be generalized to a wider group of violinists. More research is needed regarding factors influencing healthy performance, before conservatoires and professional orchestras can develop programs for a healthy playing environment for pre-professional and professional violinists.

## Introduction

Musculoskeletal complaints and pain are very common in musicians (Zaza, 1998; Silva et al., 2015; Kok et al., 2016; Viljamaa et al., 2017). Multiple studies indicate that string players have a higher risk of musculoskeletal problems, compared to other instrument groups (Fishbein et al., 1988; Davies and Mangion, 2002; Paarup et al., 2011; Rensing et al., 2018). In a large-scale study of more than 2,000 orchestra musicians, 76% indicated that they had experienced a medical problem that affected their performance during their lifetime. Of the string players in this sample, 84% had experienced at least one medical problem (Fishbein et al., 1988). Among the population of pre-professional and professional classical violinists, neck and shoulder complaints appear to be most common (Fishbein et al., 1988; Kok et al., 2015; Steinmetz et al., 2016; Viljamaa et al., 2017; Rensing et al., 2018).

Playing the violin involves a complex interaction of different factors, including posture, technique, muscle load, repetitive movements and practice time, all of which may contribute to physical complaints (Kok et al., 2018; Rensing et al., 2018). Violinists use an asymmetrical posture to play their instrument, holding their instrument between their chin and shoulder. Moreover, for the purpose of bowing, the violinist’s right arm needs to be elevated. Research shows that instrumentalists who play with an elevated arm for more than 3 hours per workday have a higher prevalence of neck and shoulder complaints (Nyman et al., 2007). In addition, Spahn and colleagues showed that specific movement patterns while playing in different positions, e.g., standing and sitting oriented to the left or right side of the music stand, result in different weight distributions. Moreover, they found that sitting in front of the music stand or oriented to the right of the music stand caused more restriction in movement of the right arm, compared to other positions. Therefore, the authors discuss postural differences that may play a role in potential health complaints (Spahn et al., 2014). Furthermore, a sudden increase in playing time, which may be experienced by students entering a conservatoire, appears to be associated with more playing-related pain (Robitaille et al., 2018). Identifying all of these different factors and how they are related can provide insights into ways of ensuring healthy performance and therefore minimizing injury risks.

Physical health problems affect violinists’ performance and the consequences can be severe (Steinmetz et al., 2016; Kochem and Silva, 2017; Kok et al., 2018). Violinists may need to adapt their playing style or they may be forced to stop playing entirely (Hansen and Reed, 2006; Kok et al., 2016). According to the injury prevention model of van Mechelen (1992), it is important to identify factors and mechanisms associated with physical health and injuries to develop preventive measures (van Mechelen, 1992). One example of such a preventive measure is a physical screening tool. A physical screening tool could be used as an injury risk management strategy to provide musicians with insights into their physical characteristics and identify areas of attention that may lead to a complaint or injury (Mottram and Comerford, 2008; Ackermann and Driscoll, 2010; Clark et al., 2013).

The number of quantitative studies focusing on possible risk factors and preventing physical health problems in violinists is currently growing (Ackermann and Adams, 2004; Park et al., 2012; Spahn et al., 2014; Smithson et al., 2017; Kok et al., 2018). In contrast to quantitative studies, qualitative studies in health research offer an in-depth understanding of health experiences and what they mean to the participants. In addition, qualitative findings facilitate the identification of contextual influences (Jack, 2006). There is only a handful of qualitative studies investigating physical health in musicians (Guptill, 2011; Schoeb and Zosso, 2012; Perkins et al., 2017; Ajidahun et al., 2019; Roos et al., 2021). A qualitative study using focus group discussions explored the implementation of exercise-based injury prevention programs for string players (Ajidahun et al., 2019). The participants in this study, including musicians and music tutors, suggested scheduling dedicated time for exercise activities within the program of the music school, rather than offering these activities in the one-to-one teaching sessions, since they are usually only 30 minutes long. In terms of compliance, the researchers found a willingness among string players to explore injury prevention programs to prevent health problems. Another qualitative study, by Perkins and colleagues, explored enablers and barriers to optimal health among music students, with a specific focus on the institutional context. In this study, three major themes were identified as enablers and barriers, namely lifestyle, support and environment. It was therefore concluded that health promotion and support within the conservatoire are important and that further investigations need to pinpoint how the culture in conservatoires can optimally facilitate students’ health and wellbeing (Perkins et al., 2017).

To the best of our knowledge, no qualitative study focusing on healthy performance in pre-professional and professional classical violinists has been carried out to date. Therefore, the research focus of this exploratory study was to gain initial insights into factors influencing healthy performance for pre-professional and professional classical violinists.

## Methodology and Methods

### Research Framework

This study is underpinned by an interpretivist research paradigm (Bunniss and Kelly, 2010). In terms of ontology and epistemology, this means that there is not one ultimate “truth”, but there are various ways of interpreting reality and knowledge is subjective. The methodology of this kind of research paradigm focuses on interpretations of participants and knowledge is constructed in the interaction between the participant and researcher. Furthermore, this perspective aims to “build a detailed picture of how a particular phenomenon is understood by those who have personal experience of it” (Bunniss and Kelly, 2010). For this epistemological approach, semi-structured interviews are a useful tool to be able to adapt during the interview and stay close to the issues that matter to the participants.

In terms of researcher reflexivity, it is important to be aware of the first author’s background in human movement sciences, music and involvement in a health department at a conservatoire. Two other researchers were consulted to discuss the analysis. They have backgrounds in human movement sciences, sports psychology and dance research and philosophy, educational research and qualitative research.

### Design and Participants

The research focus of our study was to gain initial insights into factors influencing healthy performance for pre-professional and professional classical violinists. This exploratory study was conducted using semi-structured interviews with six participants. The qualitative study design enabled us to study the participants’ perceptions of healthy performance of the pre-professional and professional violinist. Furthermore, this research design allowed us to study the participants’ views on the contextual influences associated with healthy performance (Jack, 2006).

For our study, we invited seven participants to take part in a one-to-one semi-structured interview between February and July 2020. Six participants agreed to participate. One further participant was interested but the interview ultimately did not take place due to scheduling difficulties. The participants were recommended by professional musicians and professionals in music education within the first author’s network (a manager and teacher within a conservatoire and a physiotherapist). To gain insights into different perspectives, we implemented data triangulation, whereby we invited participants with different and even multiple backgrounds. Other inclusion criteria were experience with professional or pre-professional classical violinists in terms of physical or mental health or experience as a professional or pre-professional classical violinist and an interest in the development of a physical screening tool for these violinists. Based on these inclusion criteria, we included two classical violin students, one classical violin teacher, two health professionals (one of whom graduated as a professional classical viola player) and one professional classical violin player (who is also a performance coach for musicians). All participants were female and gave their written informed consent in which they consented to the audio-recording of the interview and made clear that they understood the aim of the study. The participants’ characteristics can be found in Table 1. The study was approved by the Medical Ethics Committee (MEC-2019-0163) of the Erasmus MC University Medical Center Rotterdam, Netherlands.

**TABLE 1:** Characteristics of the participants.

Participant	Age	Background	Language during the interview
P1	45	Violin teacher (classical), professional violinist in a symphony orchestra	Dutch
P2	33	Health professional (involved in health department for musicians), graduated as a professional classical violist	Dutch
P3	24	Violin student (Master of Music, Classical Music)	Dutch
P4	24	Violin student (Master of Music, Classical Music)	English
P5	56	Professional classical violinist in different symphony orchestras, performance coach for musicians	Dutch/English
P6	34	Health professional (manual physiotherapist), with extensive experience in working with musicians*	Dutch

*This interview recording failed and we summarized the results after the interview took place, as described in the “analysis” subheading.

### Interviews

We conducted semi-structured interviews to ensure both structure and flexibility in response to the participant and to offer space for the participant’s individual expressions (Kallio et al., 2016). Semi-structured interviews allow for open-ended responses from participants for more in-depth information and encourages two-way communication. This enabled us to identify issues that matter to the participants in relation to the focus of the study. Two of the interviews took place face-to-face. Due to COVID-19 travel restrictions, we were forced to organize four interviews online, *via* Zoom (3) and WhatsApp video-calling (1). The mean duration of the interviews was 31 minutes (range: 22–53 minutes). At the start of the interview, there was time for the first author and the participant to introduce themselves. Subsequently, the first author explained the nature of the study. The interview schedule comprised four main questions focusing on physical health. The first three questions were exploratory, while the last question was direct:

(1) Which physical aspects are important in your profession/the profession of the violinist/studies?
(2) Which injuries often occur in pre-professional/professional violinists?
(3) What kind of preventive measures do you/other violinists/violin students take?
(4) Highlighting one possible preventive measure: what would a physical screening tool for violinists look like in your experience?

### Focus of the Study

The initial focus of this study was to collect input from a variety of stakeholders on how a physical screening tool could contribute to healthy performance for violinists and which aspects should be included. However, the interviews provided us with richer information than expected. The participants mentioned a variety of factors beyond a physical screening tool that were relevant to them and according to them played an important role in ensuring healthy performance for the pre-professional and professional classical violinist. To do justice to what participants identified as relevant, and in line with the interpretivist research paradigm, we broadened the focus of the study during the analysis to describe factors influencing healthy performance for pre-professional and professional classical violinists, according to the participants in this study.

### Analysis

All interviews were audio recorded. After one interview, it emerged that the recording had failed. In this case, the first author summarized the interview results immediately after it concluded and sent the summary to the participant to add any necessary information. All other interviews were transcribed verbatim.

We used thematic analysis to analyze the transcribed interviews (Braun and Clarke, 2006). We performed one analysis for the responses to the first three interview questions. In addition, we performed a separate analysis for the last interview question regarding the physical screening tool. The analyses were performed in English and Dutch quotes selected for inclusion in the current manuscript were translated professionally. The first author read all of the transcripts for familiarity. Using Atlas.ti 9, we generated initial codes. In this process similarities and differences with existing codes were considered through constant comparison. After this, we discussed how the codes could be clustered into an overall theme and relationships between the initial codes were investigated. The next step was to review the themes. In this process, we performed regular checks for a clear distinction between themes, while maintaining coherence within a theme (internal homogeneity and external heterogeneity) (Braun and Clarke, 2006). During the last step of the process, we identified the final themes. The second author and an additional researcher provided support in the analysis and discussed the codes and themes with the first author. This resulted in the rewording of some of the codes and connecting codes to more than one theme, resulting in overlapping themes. Furthermore, the subcategory “participants” recommendations’ was defined.

## Results and Discussion

The first analysis resulted in six themes derived from 106 codes representing multiple factors in fostering healthy performance for pre-professional and professional classical violinists. The themes were: (1) physical aspects (involved in playing the violin); (2) practice routine and techniques; (3) interaction between physical and mental aspects; (4) culture; (5) role of the main subject teacher; and (6) preventive measures. Furthermore, we identified 20 codes in response to interview question 4 regarding the contents of a physical screening tool. Table 2 presents the results of these analyses, including all codes and themes, as well as codes contributing to more than one theme, implying overlapping themes. Below, we first discuss these six themes as a result of our exploratory interview questions, before we present and discuss the results regarding our direct question regarding a physical screening tool. Alongside descriptions of their current situation, participants also mentioned a number of recommendations they considered useful in healthy performance. The descriptions and recommendations below all apply specifically to pre-professional and professional classical violinists.

**TABLE 2:** Themes and codes, each theme represents a cluster of codes and some codes contribute to more than one theme.

	Theme: Physical Aspects (involved in playing the violin) (PA)	Theme: Playing routines and technique (PRT)	Theme: Interaction between physical and mental aspects (IBPM)	Theme: Culture (C)	Theme: Role of main subject teacher (RMST)	Theme: Preventive measures (PM)	Theme: Physical screening tool (PST)
	**Unique codes (20):** * Muscle endurance * Muscle strength * Stamina of core * Physically optimal teaching programs not always possible * Stiff or hypermobile * Alternate standing/sitting * Cooling down * Suboptimal posture * Injuries: arm complaints, back complaints, hand complaints, low back problems, neck complaints, shoulder complaints (general, cuf tear, frozen shoulder, scapuladiskinesia, subacromial pain), wrist complaints	Unique codes (11): * Proper playing technique * Orchestra setting; warming up is important; enough playing space is not always available * Study/practice skills: proper scheduling provides rest; start playing slowly when practicing; change focus on regular basis; set playing goal *Develop own way of practicing * Different opinions about optimal technique * Difficult to change practicing habits	Unique codes (7): * Awareness * Many worries, no time for body awareness * Physical tension under pressure * Stress cause of physical complaint * Too tired for muscle strengthening exercises * Underlying factor of tension is fear * Change way of thinking/approach before working on physical aspects	Unique codes (13): * Orchestra setting; open culture needed; top down hierarchy still common; everyone is important * Awareness: musicians do not listen to their bodies; economic considerations; creates stress and pain * Development as human being, not only musically or technically * Play enough hours is important * Musician did not speak about complaints years ago * Admitting that you have a complaint means admitting that you are not good enough * Every violin players has/had complaints * Fear is taboo; creates stress and pain	Unique codes (9): * Education in cooperation * Speak out expectations * Find suiting teacher * Collaboration between MST and HP needed for optimal performance * Emphasis on mistakes; cause of fear * MST looks at posture student from own experience * MST supports adaptation of studying * Training about body	Unique codes (20): * Awareness: more body awareness is needed; instrument specific exercises to increase commitment; music students are careful; protect only fingers; increases due to injury * Breaks/recovery time is important * Enough sleep * HP (Health Practitioner); support of physiotherapist; mensendieck therapist * Stamina/endurance avoid injuries * Alexander Technique * Affordance: shoulder and chin rest should be properly adjusted * No awareness about the body in violin students * Violin players are more aware of arm than back * Orchestra setting: rehearsals are too long; suboptimal build up; change posture during rehearsal * Sports/staying fit; not many students do exercises or sports; speeds op revalidation process	Unique codes (20): * Adjustments chin/shoulder rest * Check core stability * Check injury history * Check neck area * Check (relaxed) playing posture * Check shoulders * Check scapular dyskinesis * Check arms * Check body shape of violinist * Check upper back * Check adhesions of muscles * Hypermobility * Measuring muscle strength; shoulder strength; strength hands/fingers * Mobility: neck; shoulder; wrist * Playing experience * Questionnaire including possible risk factors * Repeat screening for monitoring
**Theme: Physical Aspects (involved in playing the violin) (PA)**		Overlap PRT and PA (3): * Relaxed playing technique * Relaxed seat before playing * Affordance	Overlap IBPM and PA (1): * Different causes of injuries (also mental)	Overlap C and PA (1): * Physical pain is part of the game	Overlap RMST and PA (1): * Physical corrections of MST	Overlap PM and PA (3): * Create free movement for optimal performance * Create relaxed posture for optimal performance * Practice relaxed shoulders	
**Theme: Playing routines and technique (PRT)**			Overlap IBPM and PRT (1): * Playing with tension due to pressure to do it right		Overlap RMST and PRT (1): * Technique teacher not always optimal for student	**Overlap PM and PRT (5)**: * Warming up; to losen/stretch muscles; warming up with instrument * Integration rehearsal or lesson * Strengthen muscles should be included in rehearsal	
**Theme: Interaction between physical and mental aspects (IBPM)**				Overlap C and IBPM (2): * Keep going no matter what is not the right mentality for healthy performance * High pressure violinists - lots of notes		Overlap PM and IBPM (2): * Mental assessment * Mindfulness	
**Theme: culture (C)**					Overlap RMST and C (3): * Student should be allowed to explore own (artistic) development * Students experience fear to make mistakes which leads to insecurity * Student’s mistakes reflect on personality	Overlap PM and C (2): * Not much attention for endurance/strength to avoid complaints * Students avoid activities that involve a lot of movement	
**Theme: Role of main subject teacher (RMST)**						Overlap PM and RMST (1): * Collaboration MST/HP: decrease complaints	

*PA, physical aspects; PR and T, practice routines and technique; IBPM, interaction between physical and mental aspects; C, culture; RMST, role main subject teacher; PM, preventive measures; PS, physical screening.*

### Theme 1: Physical Aspects (Involved in Playing the Violin)

All participants mentioned their experience with injuries and complaints, or talked about those of other violinists with whom they were acquainted. They indicated that most of these injuries were located in the upper extremities. All participants reported at least two of the regions of the body that were affected: arms, back, hands, neck, wrists, and shoulders.

“…in the case of the violin this is very clear, and I know from physiotherapists, they know exactly what kind of instrument you play… when they are a bit settled in the musical world. Yes, in our case it concerns the shoulders, neck, arms…” (P1)

These findings are in line with other studies focusing on complaints in high string players and violinists (Kochem and Silva, 2017; Viljamaa et al., 2017; Kok et al., 2018; Rensing et al., 2018).

A number of physical aspects that facilitate healthy performance could also be linked to the theme of “practice routine and technique”, such as alternating sitting and standing and assuming a relaxed sitting posture before playing. A study by Spahn and colleagues exploring different standing and sitting positions for violinists, had already suggested the possible relationship between playing positions and health problems. Among others, the authors recommended considering varying different playing positions to ensure flexibility in the body (Spahn et al., 2014). Implementing different playing positions in practice, such as standing and sitting, or in rehearsals by regularly switching places in front of the music stand, would be recommended.

Specifically for violin students, the participants considered their teacher’s corrections in terms of physical aspects during playing as essential.

“…. Just now I had this during my lesson… that I used too little of the bow, so he says: ‘use a bit more bow’ and then you feel like: ‘Oh this feels nicer’, more chill to just bow relaxed.” (P3)

Participants reported that it was not always possible to organize an educational program that is physically optimal for music students. They acknowledged that students are subject to long days which include extended durations of playing. According to the participants, this suboptimal schedule also appears to result from a variety of teachers being involved in the conservatoire’s program, which makes it challenging to develop a physically optimal program for the students. Furthermore, the participants also noted aspects of the violin itself that are difficult to adapt for achieving healthy performance and may create physical complaints, such as asymmetrical properties. This so-called “affordance” of the violin refers to the actions and body postures whereby the properties of the violin enable or constrain the player to perform (Mooney, 2011; Chong and Proctor, 2020). An asymmetrical, suboptimal posture needs to be adopted to be able to play the violin. This may result in more physical health problems (Davies and Mangion, 2002). One of the participants described the long days and affordance of the violin as causes for physical problems:

“After a certain amount of hours you will experience problems. It is not a natural posture…” (P1)

#### Participants’ Recommendations

Alongside descriptions of their current situation, participants reported a number of needs and recommendations concerning physical facilitators that they felt were necessary for healthy performance. More attention to muscle endurance and muscle strength in the upper extremities and core was considered essential by the participants to support the repetitive and “unnatural” asymmetrical movement that is inherent to violin playing. Participants in both the conservatoire and orchestra contexts mentioned this:

“So yeah, what is important… is that the muscles are strong enough to keep a wrong posture for so long. That’s it basically. You cannot say that playing the violin is natural. That’s simply not the case.” (P1)

A study by Andersen et al. (2017) showed that more than half of participating professional orchestra musicians experienced a positive impact on playing from undertaking both strength training and a general fitness program. Based on these findings, we recommend investigating ways to integrate exercises or education in the students’ education or orchestra rehearsals.

In addition, the participants indicated a relaxed posture, especially relaxed shoulders and hands, and free movement as necessary for healthy performance, which emphasizes the connection with the theme of “preventive measures”.

### Theme 2: Practice Routines and Technique

The participants highlighted different practice routines that contributed to their performance. Participants all indicated that good technique is necessary, although we found various views on what constitutes this. Some participants spoke of the essential role of the main subject teacher in this matter. While others commented on the importance of this role, they also indicated that the teacher’s technique is not always best for the student and that the teacher should understand this. Teaching methods and styles vary from one teacher to another, according to their own background and training. The teacher supports the violin student to use a good playing technique. It is very important that, along with general technical skills, the teacher should be able to identify the best technique for the student (Ackermann and Adams, 2004).

“Yes, in my first year I was taught by… who says to really put active pressure on the string… that’s a different movement then what I learnt from … who teaches me now. That you do this in a relaxed way. So really, let your elbow hang more and… more like a pulling movement than a pushing movement.” (P3)

Some participants talked about a short warm-up as part of their healthy practice routine and they mostly performed it with the instrument to warm up the hands and loosen and stretch the muscles. Participants also mentioned this in terms of prevention, which links this theme to the theme of “preventive measures”. Moreover, regularly changing their standing or sitting position while playing was another method used by the participants. They used certain study skills such as setting study goals and appropriate scheduling of rehearsals and breaks as facilitators to optimize healthy performance. According to three participants, the mental pressure to avoid making mistakes can lead to physical tension, which can be avoided or reduced by changing the manner of practice. However, they emphasized the difficulty of changing these practice routines.

“you also need to… think about how you study, what you study… whether you can clear your head … whether you are not tense in your mind and in your whole body. I also find that a very important element.” (P3)

A recent review focused on changing health-related behaviors within performing arts medicine research (Norton, 2020). In this review, the Behavior Change Wheel and what this model could mean for performing arts medicine was discussed. This model explains that a person’s behavior is influenced by Capabilities (physical and psychological), Opportunities (physical and social) and Motivation. This so-called “COM-B” model is surrounded by different contextual influences in terms of intervention functions like education and training, and policy categories such as legislation and fiscal measures (Michie et al., 2011). The Behavior Change Wheel emphasizes the importance of these contextual influences as a starting point in changing behavior. Therefore, it appears to be that, besides an intrinsic motivation of the violinist to adopt healthy practice routines, the role of the teacher, peers, and organization may be essential in facilitating healthy practice routines as well.

Moreover, most participants emphasized the challenge of maintaining healthy routines during an orchestra rehearsal. They indicated that the schedule of the program in an orchestra can be quite heavy, as rehearsals are long and the participants feel that there are not enough breaks. Some participants mentioned that due to different sizes of rooms and concert halls, and sharing a music stand, violinists need to adapt to limited playing spaces which can create a suboptimal posture or more tension in the body.

Studies have shown that different playing postures can influence different physical health aspects (Price et al., 2014; Spahn et al., 2014). Regularly switching places in front of the music stand may help to decrease the physical constraints while playing.

“… what you see in an orchestra, why there are so many complaints… is because we sit close to each other. You never have the exact amount of space that you would like, you need to make specific turns to be able to see one music stand for two people.” (P1)

#### Participants’ Recommendations

In line with the importance of a relaxed technique and posture, as already mentioned under the theme of “physical aspects”, the participants also advised integrating routines and practice skills including a warm-up during practice and the orchestra rehearsal routine to increase the opportunity for healthy performance.

“…when you look at dancers…they warm up together in which they perform these muscle strengthening exercises for the whole body. We do not have that as string players. The brass players neither, nor anywhere in the classical music world. It is assumed that you do this yourself.” (P1)

Furthermore, the development of certain study skills during the practice routine was advised, including appropriate scheduling, the use of goal-setting, and inclusion of adequate breaks. In addition, the participants recommended playing a slow piece at the start of practice. Although they emphasized that this is easier to manage in the violinists’ own practice time, two of the participants also felt that this could be applied to orchestra rehearsals. As was already mentioned in terms of a healthy study routine, the Behavior Change Wheel model in studies looking at the implementation of healthy routines, could further elaborate on how violinists can implement these healthy routines within their own practice and how these can be applied within specific contexts, including orchestras.

### Theme 3: Interaction Between Physical and Mental Aspects

Despite the fact that the questions in the interview guide mainly focused on physical health, the participants felt that this “could not clearly be separated from mental aspects” (P5) and that the interaction between the two plays an important role in healthy performance. They indicated that they sometimes experience mental pressure and stress which could result in physical tension and therefore a lower load and capacity:

“You always keep looking how to stay relaxed…, when you play difficult pieces. Making sure that you do not cramp.” (P1)

The participants also mentioned an overall tendency to avoid mistakes, which could also cause tension in the body. They described that the busy life of a musician or music student makes this even more difficult: according to them it feels that there is no time left to be aware of the body or do exercises to increase stamina and muscle strength to create a healthy balance between load and capacities:

“In our practice and in our studies, there are so many things that we have to worry about, so many things, that the body is just like… I don’t pay attention to it at all…” (P4)

These results are in line with a study by Perkins et al. (2017) addressing the notion that music students are confronted with challenges, including an intensive program, competition and performance feedback, which can result in psychological distress.

Looking at the context of professional orchestras, the work environment is psychosocially demanding and research has shown a relationship between perceived occupational stress and physical health problems (Middlestadt and Fishbein, 1988; Holst et al., 2012; Rickert et al., 2013). Besides education about stress reduction, orchestras may benefit from a positive and supportive working environment (Davies and Mangion, 2002; Rickert et al., 2013). Examples include warm-ups and rest in the workplace which could contribute to lower stress levels and fewer physical complaints.

According to the participants, one barrier related to the high pressure that violinists experience is the fact that violinists play many notes compared to other instrument groups in an orchestra setting. In addition to the importance of being physically fit, which links this theme to “physical aspects”, the mental pressure to rehearse these pieces is perceived as high by the participants:

“For example, when you need to rehearse a whole symphony for an orchestra project in a short timeframe, then I am really stressed because I need to go ‘quickly, quickly’, you know. But this is also really bad for your muscles, so you must rest.” (P3)

#### Participants’ Recommendations

The participants acknowledged that the relationship between physical and mental aspects appears to be complex. However, they did not have many recommendations for improving the situation. Two participants talked about the need to change the way of thinking or the “way of approaching” their practice first, before starting to work on physical aspects. “Keep going no matter what is not the right mentality” (P4) for a healthy performer.

Education about stress reduction may support maintaining healthy stress levels and therefore reduce physical tension (Perkins et al., 2017).

### Theme 4: Culture

Participants spoke about the importance of a culture that allows violinists to optimize healthy performance. This is consistent with the Behavior Change Wheel model in which, besides individual aspects, the importance of the context is emphasized (Michie et al., 2011; Norton, 2020).

Although it was noted that the culture is changing in a positive way [“years ago you did not speak about complaints…” (P1)], the participants indicated that there still seems to be a taboo in some environments regarding mental or physical complaints. This was also documented in a qualitative study in which musicians mentioned that they see injuries as “a sign of weakness, failure, and poor musicianship” (Rickert et al., 2014a, p. 94). The authors found that a general negative perception of injuries may result in a continuation of playing while experiencing pain.

Some participants discussed their fear of making mistakes, as previously mentioned under the theme of “interaction between physical and mental aspects”. This fear was indicated by one of the participants as being part of a culture in which music students are afraid to experiment and discover their technique and style. She illustrated this with the expression: “it feels like you are wearing a harness and walk on egg shells” (P5) while trying to perform at the same time.

An overall complex cultural issue mentioned by the participants was that they feel that pain is still accepted as part of the profession as violinist. One of the participants explained this barrier by commenting that some violinists feel that expressing a complaint feels like admitting that they are not good enough for their profession. Therefore, they “ignore it” (P5). One participant described this situation as follows:

“…Yes, it is kind of… it’s very bad but I think we try to live with it. If it’s not so bad, we are not really concerned about it.” (P4)

Three participants indicated that in their experience “there is no violinist who does not have a problem” (P1). Participants reported that they – or pre-professional or professional violinists they know - make use of support from physiotherapists or other healthcare practitioners, although in most cases they seek this kind of support when they have already been in pain for a while. The participants refer to a low awareness of listening to the body among pre-professional and professional violinists and the tendency of not recognizing or acknowledging physical complaints in a timely manner. This was implied in a previous study, in which different injury definitions in classical music students were compared. In this study, the “all musculoskeletal complaints” definition resulted in a much higher one-year prevalence (96.6%) than the “medical attention” definition (17.2%), indicating that music students do not easily seek help (Steemers et al., 2020). From these findings, it seems that offering support from healthcare practitioners with a focus on prevention may result in earlier detection of possible injuries.

One of the participants mentioned that there is a very difficult balance between creating healthy performance on the one hand and the notion that “if you do not practice so much… you will not make it as a musician”(P1) on the other hand.

#### Participants’ Recommendations

This theme could be connected with the “physical aspects” theme. Participants indicated that there is a need for bodily awareness, for example, by offering endurance and strength training, to increase stamina as part of both the orchestra rehearsal and the curriculum at the conservatoire. Several studies have recommended further examination of the cultures of conservatoires and investigations into ways to increase health in the music student population and foster a supportive learning environment (Papageorgi et al., 2010; Perkins et al., 2017). Future research should focus on injury-prevention measures and healthy performance within the curriculum. This may facilitate a more open culture in which pre-professional musicians talk about their complaints and make health awareness a more natural part of healthy performance.

The participants suggested that the main subject teacher could also play a role in this open environment during the teaching process by inviting the student to explore their own style and technique. Therefore, the theme of “culture” is connected to the “role of the main subject teacher”. In the context of the professional orchestra, this open environment appears to be important for creating a low threshold to discuss complaints, focus on prevention, and, if necessary, find support. Support from orchestra management in injury prevention as part of the rehearsals is important for lowering this threshold (Rickert et al., 2014b). Based on these findings and the results in our study, it appears to be that the orchestra management may also play a role in emphasizing the importance of being healthy to optimize performance.

In general, the current study uncovered the need of the participants, in both conservatoires and the professional context, to start a cultural change in which it feels natural to focus on prevention and bodily awareness as part of healthy performance and to open up about physical and mental complaints. More studies focusing on cultural and behavioral change in these contexts would be of tremendous value to further investigate this need.

### Theme 5: Role of the Main Subject Teacher

Participants indicated that the corrections of teachers are essential for fostering healthy performance. The main subject teacher is the “first point of contact” (P6) for the student. Both of the student participants mentioned that their main subject teacher supported them in incorporating a more relaxed playing posture and adapting their way of studying and practice routines if necessary. A previous study demonstrated that students tend to seek help from their teachers when experiencing playing-related complaints, who are therefore often the first point of contact (Ioannou and Altenmüller, 2015). Moreover, the participants felt that teachers can encourage students to include certain practice routines and provide specialized practice strategies adapted to the piece that the student is rehearsing. According to Blackwell et al. (2020), vitality in music students seems to be dependent on the quality of their relationship with their teacher, which emphasizes the importance of this relationship in healthy performance.

However, it was also mentioned by the participants that teachers judge posture and practice routines from the perspective of their own experience, and that it is important for teachers to acknowledge that their way of playing is not always the most optimal for the student. Some participants indicated that the main subject teachers may benefit from training about the body and bodily awareness.

“I would say to also to… to involve the main subject teachers in these things. I mean, if you ask a violinist who never received this type of education, you will not pass this on to your students and I understand that. It would be nice to hear something from the main subject teacher about the body.” (P4)

We found overlap between the themes of “role of the main subject teacher” and “culture”. As mentioned under the theme of “culture” by the participants, the main subject teacher also plays an important role in creating an open environment, ensuring that the student is allowed to explore, find their own playing style, and is not afraid to make mistakes. A doctoral thesis by Norton (2016) provided more information on the teacher’s perspective on health promotion. In this doctoral thesis, teachers acknowledged their role in the health promotion of their students by, among others, discussing health-related topics with their students to support them in preventing injuries. One of the participants mentioned that the main subject teacher occasionally over-emphasizes mistakes, instead of offering compliments concerning what is going well and discussing skills and techniques that can be developed. According to her, in this way, the fear of making a mistake increases.

“The main focus is on things that are not going well. And if you only focus on that part, then you only see what can go wrong. Which causes fear. Instead of, okay, I can do this, and that, and that well. The other things, I need to develop.” (P5)

Overall, the participants stated that the role of the main subject teacher appears to be crucial in both supporting the student in the most optimal practice routine and as first point of contact in health-related questions and preventing injuries. Therefore it seems important that the teacher has knowledge about injury prevention and knows how to apply this within their teaching routines and also has a sense of when and how to refer the student to healthcare practitioners for support.

#### Participants’ Recommendations

It was suggested that the main subject teacher and health specialists should collaborate to optimize the student’s performance. According to the participants, such a collaboration facilitates further commitment to engaging in healthy performance behavior by the student. This was also recommended by Norton (2016), although respondents in this research expected difficulties of such a collaborative health team because of little direct contact between the various representatives. We feel that a team of healthcare practitioners should be integrated as part of the organization which would substantially increase the level of cooperation. Good integration within the context of the conservatoire or orchestra and communication between the different persons involved in this team seems key, as was also indicated by one of the participants:

“I think that when you do this together… that it certainly has added value in comparison to when one of the two tries to make adjustments. Because when only the physiotherapist makes the adjustments, then he or she does not know the desires of the teacher properly to judge the qualities and effect of the playing technique and then you see that such an intervention or adjustment is not fully supported by the main subject teacher and in the end therefore also not by the student.” (P2)

### Theme 6: Preventive Measures

The participants used different tools and strategies to facilitate healthy performance. They used warming up on the violin to loosen or stretch the muscles before playing or rehearsing, for example, by playing scales and starting with a slow piece of music. Furthermore, methods such as the Alexander Technique seem very popular with pre-professional and professional violinists. The Alexander Technique is an educational method that addresses identifying harmful habits built up over time and learning to move more freely by developing more specific awareness of bodily use (Tarr, 2011). Mindfulness was also highlighted as a tool to provide physical and mental relaxation.

Although more high-quality research is necessary to draw firm conclusions about the effects of these methods on healthy performance, some studies have already indicated initial positive results. The Alexander Technique may contribute to reducing playing-related pain and improving music performance in musicians (Davies, 2020). Mindfulness also appears to have a beneficial effect on the stress levels of music students (Bartos et al., 2021). Moreover, as recommended under the themes of “physical aspects” and “culture”, the participants suggested considering health promotion with an emphasis on bodily awareness, including instrument-specific exercises, for both conservatoires and orchestras.

Bodily awareness was noted as an important facilitator in prevention. Participants reported that in general, violinists lack bodily awareness or are only aware of specific body parts. One participant even stated that violin players are not aware of the body at all. The participants indicated that bodily awareness increases when experiencing an injury.

“We are really not conscious about this… really, I mean, I think for violin players, I would say for musicians in general, but for violin players it is the least important thing, the body. I mean, it’s very important, it’s the basic, if you don’t have a body you cannot play but… but for us it’s… because we have to worry about so many things.” (P4)

Other studies have already shown that, health promotion can foster a healthier study environment and working conditions. Education and interventions at conservatoires usually focus on addressing physical and mental health problems, rather than preventing and coping with the challenges of making music (Araújo et al., 2017). It has therefore been recommended that conservatoires should equip their music students with the knowledge and tools regarding health issues, preferably at the start of the program, when students will most likely begin to increase their playing hours (Brandfonbrener, 2009; Kochem and Silva, 2017; Perkins et al., 2017). Considering the number of complaints, awareness of physical and mental health seems essential in both professional and pre-professional violinists to facilitate healthy performance. Based on our results and previous findings, conservatoires may consider Alexander Technique, Mindfulness or instrument-specific exercises as part of their curriculum. These tools can be useful throughout the students’ studies and professional career of the violinist.

In terms of sports and staying fit, the participants indicated that not many violinists engage in regular physical exercise. However, according to one of the health specialists, sports and exercise speed up the recovery process.

“It is noticeable that students who do sports or exercise recover faster and are less likely to develop new complaints.”(P6)

#### Participants’ Recommendations

Participants suggested that an increase in bodily awareness is needed for healthy performance and the use of instrument-specific exercises could increase commitment. Therefore, the theme of “preventive measures” is closely connected to “physical aspects”. According to the participants, there should be at least a focus on stamina due to the long hours that violinists endure, as well as the fact that violinists play many notes, especially during orchestra rehearsals. Participants also reported the influence of taking sufficient breaks during recovery. As such, this theme could also be linked to “practice routines and technique”.

“My personal experience as a violinist is that stamina is something that is important and a lot of people struggle with it.” (P2)

Furthermore, participants advised receiving support from a variety of healthcare practitioners; for example, a physiotherapist, Alexander Technique teacher or Mensendieck therapist who could support the violinists and increase awareness of bodily movements while playing, such as the shoulder area and good adjustment of the chinrest. Mensendieck therapy offers postural exercises to prevent musculoskeletal complaints. Baadjou et al. (2017) investigated the relationship between body posture, muscle activity, and sound quality. They hypothesized that the postural exercise therapy, guided by a Mensendieck therapist, would influence body posture and muscle activity and therefore increase the sound quality. The results of this study showed that muscle activity may be changed by the exercises but no relationship between posture and sound quality was discovered (Baadjou et al., 2017).

Besides support from healthcare practitioners, the participants implied that collaboration between the main subject teacher and a health specialist could optimally support violin students in both health and creative aspects of an optimal performance.

### Physical Screening

**FIGURE 1:**
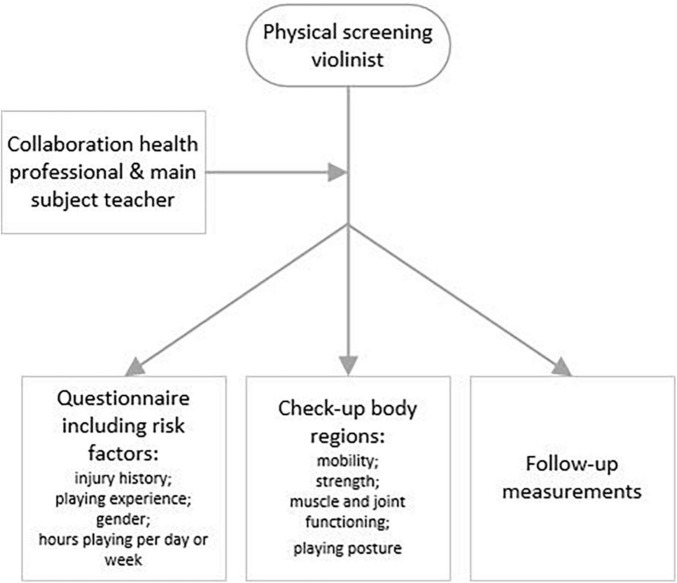
Aspects that should be included in a physical screening tool for violinists according to the participants.

The participants were explicitly asked about their thoughts regarding a physical screening tool for violin students (research question 4), as this was our initial research focus. The physical screening tool was presented as a preventive tool for pre-professional violinists to offer them insights into their strengths and areas for further development. Participants emphasized that multiple factors contributed to their complaints. Therefore, they felt that this variety of factors should be included in the physical screening tool, including various regions of the body, the inclusion of a questionnaire, follow-ups, and collaborations between health professionals and the main subject teacher. These factors are depicted in Figure 1.

All participants mentioned that in the case of pre-professional violinists, specific body regions should be checked by a physiotherapist or other healthcare practitioner. These body regions included the neck area, the arms, the wrists, the hands and fingers, the upper back and the shoulders. Regarding these regions of the body, they reported that the healthcare practitioner should look at different muscle and joint functions, muscle strength, mobility, and playing posture.

These findings are in accordance with a study by Clark et al. (2013) who already advised tailoring screenings to specific instrument groups. Araújo et al. (2020) developed a fitness screening protocol for the general population of music students including mobility and strength exercises, among others. They advised musicians to strengthen their supportive musculature and be aware of strength imbalances. Therefore, we suggest that a physical screening tool, including strength tests, should be specifically tailored to violinists to increase understanding and commitment among participants of the screening.

Some participants suggested including a questionnaire as part of the screening. According to them, this questionnaire should be completed before the start of the screening and it should “include risk factors for muscle and joint complaints” (P2); for example, injury history, gender, playing experience, and the number of playing hours per day or week. van Seters et al. (2017) have already successfully investigated this mixed approach by using screening tests and questionnaires to look into risk factors for lower-extremity injuries among dance students.

Three participants indicated that a collaboration between a health specialist and the main subject teacher is important in terms of commitment to this screening tool for violin students. The main subject teacher is the first point of contact for the student regarding their violin playing, as well as the person who can correct and support the student to play as optimally as possible. Furthermore, to gain more insights into possible risk factors, participants advised in favor of repeating the screening after some time to more closely monitor the violinist.

#### Practical Implications

The current study was carried out to gain some initial insights into factors influencing healthy performance for pre-professional and professional classical violinists. It is important to emphasize that the current study involved a small, selected group of stakeholders and the practical implications described below should therefore be considered as initial, exploratory insights rather than firm conclusions. Nevertheless, data revealed some practical implications to increase healthy performance, which can be investigated in future research:

• To prevent injuries, it may be worth using different playing positions when practising, such as standing and sitting, and changing places in front of the music stand when rehearsing.

• To increase stamina, exercises to increase muscle strength and endurance could be taught and included in orchestra rehearsals.

• Participants also recommended integrating practice routines including appropriate scheduling, warm-ups, adequate breaks and starting rehearsals or practice with a slow piece. Integrating these could create a supportive environment in terms of prevention and could contribute to fewer physical complaints and lower stress levels. Education about stress reduction may also contribute to maintaining healthy stress levels and reducing physical tension.

• The main subject teacher plays an important role as first point of contact in supporting the student in developing a good and healthy technique, but also in terms of physical complaints and prevention. Teachers may benefit from a body awareness training and increase their knowledge about the process of referring the student to a healthcare practitioner.

• To increase collaboration between main subject teacher and healthcare practitioners, it may be recommended to integrate a team of healthcare practitioners with different focus points within the organization of the conservatoire or orchestra.

• A focus on injury prevention and healthy performance as an integral part of the curriculum may facilitate a more open culture in which musicians talk about their complaints and make health awareness a more natural part of healthy performance.

• Alexander Technique, Mindfulness, and instrument-specific exercises to increase commitment could be useful tools throughout the studies and future career of the violin students.

• A physical screening tool may be a useful preventive measure: it could include check-ups of mobility, strength, muscle and joint functioning, and playing posture. Moreover, a questionnaire regarding injury history and playing experience could be completed before the screening takes place. Follow-up measurements and a collaboration of the health practitioner with the main subject teacher may contribute to increased commitment and optimize monitoring healthy performance of the violinist.

### Strengths and Limitations

To our knowledge, this is the first exploratory study focusing on healthy performance in pre-professional and professional classical violin players. Using data triangulation by including participants with different expertise and backgrounds provided us with many potentially useful insights into factors influencing healthy performance in violinists.

Using semi-structured interviews ensured both structure and flexibility during the interview process. Although the interview questions were focused on physical health, the interaction between physical and mental health appeared to be essential in healthy performance. Due to the nature of semi-structured interviewing, we were able to explore and adapt our questions during the interviews and the interaction between physical and mental aspects became an important theme. While statistical types of generalizability are difficult to apply to qualitative research, analytical or “vertical” generalizability may be provided. These types of generalizations are not based on fixed, objective data, but rather on fluid ideas and interpretations of participants (Smith, 2018). However, our study sample is small and includes only females. It should therefore be considered as an initial, exploratory study offering some first insights without drawing firm conclusions. Future studies should include a larger sample and should also include male violinists in order to make sure the collected insights may be generalizable to a broader population of pre-professional and professional violinists.

The first author’s background is important to be aware of in terms of the interpretivist research paradigm considering that knowledge is generated as a result of interaction between the participant and researcher. The first researcher of this study considers healthy performance an essential topic for pre-professional and professional violinists and has some pre-existing assumptions, partly based on previous studies and literature, which are also used in the development of the interview guide. This is all accepted in the interpretivist perspective, but it is important to provide this transparency. The familiarity with the working field of musicians and health professionals may also have created a more open interview in which the participants feel that the first author understands their situation, which can thus provide more in-depth results. Moreover, we consulted with two other researchers with different backgrounds to thoroughly check and discuss the analysis.

Our study combined perspectives from music students, professionals, and health specialists to provide potentially useful information and implications for healthy performance at different points in the violinist’s career. The overall results suggest that healthy performance comprises a wide variety of factors that are closely connected, as was also presented in Table 2. The connections between themes suggest that the different stakeholders involved need to collaborate as a team around the violinist for optimal support. This warrants further research, investigating which different stakeholders could be involved to support the pre-professional or professional classical violinist on this range of aspects.

## Conclusion

Our small-scale, exploratory study, including six participants, identified six themes as important for healthy performance of pre-professional and professional violinists. The themes were: (1) physical aspects (involved in playing the violin); (2) practice routine and techniques; (3) interaction between physical and mental aspects; (4) culture; (5) role of the main subject teacher; and (6) preventive measures. Furthermore, the six participants indicated that multiple factors should be included in a physical screening tool, including various regions of the body, the inclusion of a questionnaire, follow-ups, and collaborations between health professionals and the main subject teacher. We would like to emphasize that our results are based on the opinions, attitudes, and ideas of a small, selected group of participants only and cannot be generalized to a wider group of violinists. More research is needed regarding these and other factors influencing healthy performance, before conservatoires and professional orchestras can develop programs for a healthy playing environment for pre-professional and professional violinists.

## Data Availability Statement

The datasets presented in this article are not readily available because the dataset contains medical data. Requests to access the datasets should be directed to SS, ssteemers@codarts.nl.

## Ethics Statement

The studies involving human participants were reviewed and approved by Medical Ethics Committee (MEC-2019-0163) of the Erasmus MC University Medical Center Rotterdam, Netherlands. The patients/participants provided their written informed consent to participate in this study.

## Author Contributions

SS initiated the study, performed the data collection, and wrote the first draft of the manuscript. JS participated in drafting the article. MV and JS contributed to the data analysis. MV, JS, MM, and SB-Z reviewed and edited the article. All authors gave final approval of this version to be submitted.

## Conflict of Interest

The authors declare that the research was conducted in the absence of any commercial or financial relationships that could be construed as a potential conflict of interest.

## Publisher’s Note

All claims expressed in this article are solely those of the authors and do not necessarily represent those of their affiliated organizations, or those of the publisher, the editors and the reviewers. Any product that may be evaluated in this article, or claim that may be made by its manufacturer, is not guaranteed or endorsed by the publisher.
